# 1576. Implementation of a Pharmacist-Driven Initiation of Antiretroviral Therapy in Patients Newly Diagnosed with Human Immunodeficiency Virus

**DOI:** 10.1093/ofid/ofad500.1411

**Published:** 2023-11-27

**Authors:** Kamron Amir Griffith, Katie L Wallace, Bobbi Jo Stoner, Donna R Burgess, Sarah B Jeter

**Affiliations:** Parkland Health, Dallas, Texas; University of Kentucky HealthCare, Lexington, KY; University of Kentucky Healthcare, Lexington, Kentucky; UK HealthCare, Lexington, KY; University of Kentucky HealthCare, Lexington, KY

## Abstract

**Background:**

Human Immunodeficiency Virus (HIV) infects over one million people annually. A major barrier to ending this pandemic is access to care. Prompt antiretroviral therapy (ART) initiation after diagnosis reduces time to viral suppression, decreases rates of opportunistic infections, and increases patient retention. At University of Kentucky HealthCare, there was a significant wait time from intake appointment to ART initiation with a provider. Beginning in November 2019, the HIV clinical pharmacist developed a program to initiate ART at intake appointments to curb this delay.This study was conducted to evaluate the impact of this initiative.

**Methods:**

We conducted a retrospective, quasi-experimental study assessing newly diagnosed HIV ART-naïve adult patients pre- and post-implementation of the pharmacist-driven ART initiation. Exclusion criteria included initial HIV-1 RNA < 40 copies/mL (undetectable) and patients transferring care. Those enrolled between November 2017 to October 2019 were included in the pre-group and those enrolled from November 2019 to November 2021 in the post-group. The primary outcome was time to undetectable from ART prescribing. Secondary outcomes included time to HIV-1 RNA < 200 copies/mL from ART prescribing, and time to undetectable from HIV-1 diagnosis.

**Results:**

Overall, we included 185 patients (pre-group=104 and post group=81). Undetectable was achieved in 95.5% of patients. Demographics were similar between the groups (Table 1). Median days to undetectable (63.0 vs 56.5; p=0.518) and HIV-1 RNA < 200 copies/mL (49 vs 43; p=0.388) from ART prescribing were similar between the two groups. Median days to undetectable from diagnosis (122 vs 85; p=0.004) and days to ART prescribing from initial visit (31 vs 0; p=0.001) were significantly shorter in the post-group. Median days to initial visit from HIV-1 diagnosis were shorter in the pre-group (13.5 vs 18.0; p=0.015). One-year retention was similar (93.2% vs 90.0%; p=0.433).

**Figure:**
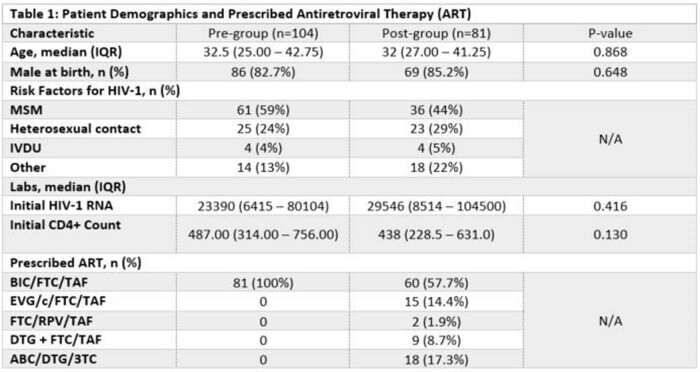

**Conclusion:**

Our study showed a significantly shorter time to undetectable from HIV-1 diagnosis with ART initiation by a clinical pharmacist at intake visits. Additionally, retention of care remained similar despite ART initiation prior to provider visit. This initiative improved timely access to care at our institution.

**Disclosures:**

**All Authors**: No reported disclosures

